# The association of food deserts with readmission and re-operation following long-segment lumbar fusion

**DOI:** 10.1007/s00701-026-06823-5

**Published:** 2026-04-11

**Authors:** Sasha Howell, Yifei Sun, Nicholas M. B. Laskay, Lucia D. Juarez, B. Grey Vandeberg, Jovanna Tracz, Anil Mahavadi, James Mooney, Jakub Godzik

**Affiliations:** 1 Department of Neurosurgery, University of Alabama at Birmingham, 1030 Faculty Office Tower, 510 20th Street S, Birmingham, AL 35233 USA; 2 Heersink School of Medicine, University of Alabama at Birmingham, Birmingham, AL USA; 3 Department of Neurological Surgery, Columbia University, New York, NY USA; 4 Division of General Internal Medicine and Population Science, Heersink School of Medicine, University of Alabama at Birmingham, Birmingham, AL USA; 5 Department of Neurosurgery, Virginia Commonwealth University, Richmond, VA USA

**Keywords:** Social determinants of health, Lumbar fusion, Food access, Nutrition, Postoperative outcomes, Health disparity

## Abstract

**Background:**

Nutrition is increasingly recognized as a key determinant of postoperative recovery in spine surgery. An estimated 17% of U.S. adults reside in a food desert, reflecting a substantial population at risk for poor nutritional status. However, the role of residence in food deserts with spinal fusion outcomes remains unknown.

**Objectives:**

We sought to investigate the association between living in a food desert and rates of reoperation and postoperative complications after long-segment lumbar fusion.

**Methods:**

We retrospectively reviewed all adult patients at a single institution from 2011 to 2023 who underwent open long-segment (≥ 4 levels) lumbar spine fusion. Patient addresses linked with residence in a food desert as defined by the Economic Research Service. Multivariate logistic regressions were utilized to assess the association of food deserts with readmissions and reoperations.

**Results:**

We identified 354 patients [median age at time of operation was 66 (IQR 59–72)] who met the inclusion criterion, of which 123 (35%) resided in a food desert. Patients in a food desert had higher neighborhood deprivation (80 vs 59, *p* < .001) and did not have differences in BMI (29.8 vs 29.9 *p* = .4). In multivariate regression analysis adjusting for frailty, clinical, socioeconomic, and operative characteristics, patients residing in a food desert had independently increased odds of re-operation within one year (OR 1.84, 95%CI 1.03–3.30) and readmission due to infection or wound breakdown (OR 3.09, 95%CI 1.03–10.1). Patients residing in a food desert were more likely to require reoperation for wound revision (8.9% vs 3.0%, *p* = .016) within one year. Using weighted survival analysis, patients who lived in a food desert had sooner times to reoperation (*p* = .045).

**Conclusions:**

Residence in a food desert may be a risk factor for reoperations following long segment lumbar spine surgery. Nutritional access may be an underlying driver of socioeconomic disparity impacting long-segment lumbar fusion.

**Supplementary Information:**

The online version contains supplementary material available at 10.1007/s00701-026-06823-5.

## Introduction

Social determinants of health (SDoH) play a critical role in individual health outcomes [35]. Factors such as race, insurance coverage, and education level have been linked to variations in postoperative satisfaction, patient-reported outcome measures (PROMs), and complication rates [5, 8, 24, 35, 43]. Significantly, there is increasing evidence regarding the impact of a patient’s built environment, defined as the socio-environmental infrastructure in which we live, work, and receive care in determining neurosurgical outcomes [10, 37]. Measures such as Area Deprivation Index (ADI) and Social Vulnerability Index (SVI) provide good overall captures of SDoH, and have been utilized to identify patient populations at increased risk for poor outcomes [12, 19, 43]. Although numerous drivers of health disparities in spine surgery outcomes have been identified, only a limited number of these metrics translate into actionable opportunities for targeted interventions.

Significantly, identifications of food deserts are an often under-recognized aspect of a patient’s socioeconomic environment. It is estimated that anywhere from 5 −17% of US citizens reside in a food desert [21]. These patients often have access to lower levels of nutritious food and are more prone to suffer from metabolic diseases, obesity, and have worse cardiovascular health outcomes, highlighting the relationship between residence in a food desert and population nutrition [17, 23, 31]. The role of proper nutrition in postoperative outcomes for recovery, wound healing, and overall rehabilitation following spine surgery is well establshed [9, 33]. Therefore, residence in a food desert may portend poor outcomes in this population. As a rapidly quantifiable measure of a patient’s built environment, residence in a food desert may offer an accurate capture of a patient’s lifestyle and nutritional status in the pre and post-operative period. However, the relationship between residence in a food desert and lumbar spine outcomes remains unexplored.

Thus, we sought to evaluate the effect of food deserts with surgical recovery, particularly readmission and reoperation following elective long-segment thoracolumbar fusion. Primary outcomes include reoperation rates at one year. Secondary outcomes included infection rates and reasons for reoperation. We hypothesized that an area-level measure of food insecurity could help identify patients at heightened risk of reoperations and complications following long-segment thoracolumbar fusion.

## Methods

This single center retrospective cohort study was conducted with Institutional Review Board approval (IRB-300009309). This article was written in compliance with the STROBE (Strengthening the Reporting of Observational Studies in Epidemiology) guidelines [40]. Patient consent was not required due to the retrospective nature of this study.

### Study design

We identified all patients undergoing thoraco-lumbar fusion from 2011 to 2023 using CPT and ICD9/10 codes from an institutional administrative database. Review of the electronic medical record was conducted to include only fusions involving ≥ 4 intervertebral. Long segment fusion was selected based on previous literature, hypothesizing that these procedures involved significant tissue disruption and required increased nutritional needs [28]. We excluded any non-elective cases. Patient demographic, clinical, and operative characteristics for each patient were collected via review of the EMR.

### Defining variables

Study variables included were age at time of surgery categorized according to standard groups (< 65, 65–75, and > 75), race (white, Black, and other), gender (Male or Female), and insurance status which was categorized as private, public (Medicare, Medicaid, Tricare), or indigent/self-pay [32]. Body mass index (BMI) at time of surgery was collected from the EMR and categorized according to current convention (< 18.5, underweight; 18.5–25, normal weight; 25–30, overweight; > 30, obesity). Frailty was calculated according to the hospital frailty risk score method [14]. Total levels fused were categorized as 4–8 levels, 8–12 levels, and 12 + levels of fusion.

Post-operative variables included were unplanned reoperations within one year of the index operation. Reasons for reoperation were determined by reviewing the operative or clinic note from the associated encounter and categorized as CSF/pseudomeningocele, instability/continued symptoms, post-operative acquired deformity, infection/wound revision, and other. Readmission at 30 days was determined according to standards set by the Center for Medicare/Medicaid, and etiologies were categorized as due to medical, wound-related, or neurological complaints [1].

Patient addresses at time of surgery were extracted from the EMR and underwent geospatial joining to area level metrics such as food desert status and neighborhood disadvantage, represented by Area Deprivation Index (ADI). Food access was retrieved from the United States Department of Agriculture (USDA) Economic Research service (ERS) Food Access Research Atlas [3]. Food deserts were defined as living in a census tract where ≥ 100 households did not have a vehicle and lived beyond a half mile from the nearest supermarket. ADI was retrieved from the Neighborhood Atlas dataset produced by the Center for Health Disparities Research at the University of Wisconsin School of Medicine and Public Health, with higher ADI indicating a higher level of socioeconomic disadvantage [26]. High ADI was defined as being the 90th percentile of national ADI percentile for the purposes of analysis [38].

### Statistical analysis

Categorical, binary, and ordinal variables were summarized as counts and percentages, and continuous variables were summarized as the median and interquartile range (IQR). Difference between groups was analyzed via utilizing the one-way analysis of variance (ANOVA), Pearson’s chi-squared test, Wilcoxon rank sum test, or Fisher’s exact test.

Multivariate logistic regressions to control for confounders was conducted to investigate the independent effect of residence in a food desert with outcomes of interest. Multicollinearity was assessed via variance inflation factors. To assess differences in time to reoperation within one year, we utilized the Flemington-Harrington weighting to conduct survival analysis to account for the uneven distribution of reoperation rates (Supplemental Digital Content, Table S1). Area under the receiver operating curve (AUROC) were utilized to assess the discrimination ability of each multivariate model to discriminate for the outcome of interest. Patients who were missing baseline data were excluded from relevant analysis. Sensitivity analysis was conducted via E-value calculation [41]. Statistical significance was set at α = 0.05**,**and all tests for significance were two-sided. All statistical analyses were performed using R (version 4.3.1, R Foundation for Statistical Computing, Vienna, Austria) [2].

## Results

### Patient characteristics and demographics

We identified a total of 354 thoraco-lumbar fusions [median age 66 years (IQR: 59–72)]. Of the cohort, 123 (35%) of the cohort resided in a food desert. The age distribution was similar across the groups (*p* = 0.4). Gender distribution was balanced, with 48% female and 52% male participants in the overall cohort. Racial demographics included 14% black participants, with no significant differences observed between groups (*p* > 0.8). The median BMI was 29.8 kg/m^2^ (IQR 26.6–33.7), with 34% of patients being considered overweight and 49% being obese. In total, most of the cohort received between 4–8 fused intervertebral levels, with 3.1% between 8–12 and 2.3% having greater than 12. Of the procedures, most (71%) were posterior fusions, while 29% of the procedures included a combined approach. Of the cohort, 21% of patients had at least one reoperation within one year. Reoperations within the first year were most often due to instability/continued symptoms, followed by wound breakdown and acquired post-operative deformity. Further characteristics are detailed in Table 1.

**Table 1:** Patient characteristics

Characteristic	Overall
	*N* = 354^*1*^
Age	66 (59, 72)
< 65	164 (46%)
65–75	144 (41%)
> 75	46 (13%)
Gender	
Female	170 (48%)
Male	184 (52%)
Race	
White	300 (85%)
Black	48 (14%)
Other	6 (1.7%)
Smoking status	
Never	183 (52%)
Former	118 (33%)
Current	53 (15%)
Frailty	
Low Risk	210 (59%)
Intermediate Risk	97 (27%)
High Risk	47 (13%)
BMI category	29.8 (26.6, 33.7)
Normal weight	57 (16%)
Underweight	1 (0.3%)
Overweight	122 (34%)
Obese	174 (49%)
Levels of Fusion	
4–8	335 (95%)
8–12	11 (3.1%)
> 12	8 (2.3%)
Surgical Approach Type	
Combined	101 (29%)
Posterior	253 (71%)
Insurance Category	
Private	114 (32%)
Public	238 (67%)
Indigent/Self Pay	2 (0.6%)
Food Desert Residence	123 (35%)
Had any 30-day Readmission*	60 (17%)
Readmission 30-day Etiologies**	
Medical	20 (33%)
Weakness/Pain	25 (42%)
Wound/Infection	15 (25%)
Had any Reoperation within One Year*	74 (21%)
Reoperation Reasons within one year**	
CSF/Pseudomeningocele	5 (1.4%)
Instability/Persistent symptoms	40 (11%)
Post-op deformity	10 (2.8%)
Infection/Wound Revision	18 (5.1%)
Other	5 (1.4%)
Time to Reoperation	51 (19, 181)

^*1*^ n (%); Median (Q1, Q3)

^*2*^ Wilcoxon rank sum test; Pearson’s Chi-squared test; Fisher’s exact test; Wilcoxon rank sum exact test

*Only the first event within time period counted

** subcategories/etiologies counted if they occurred at any point within the time period, with multiples allowed

Patients in a food desert had higher neighborhood disadvantage (80 vs 59, *p* < 0.001), and had greater rates of readmission due to wound or infection compared (8.1% vs 2.6%, *p* = 0.012), and were more likely to require reoperation infectious washouts or wound revisions within the first year (8.9% vs 3.0%, *p* = 0.016) (Table 2).

**Table 2:** Patient comparisons by food desert status

Characteristic	Food Desert		*p*-value^*2*^
	No	Yes	
	*N* = 231^*1*^	*N* = 123^*1*^	
Age (years)	66 (60, 72)	65 (59, 72)	0.4
< 65	101 (44%)	63 (51%)	
65–75	99 (43%)	45 (37%)	
> 75	31 (13%)	15 (12%)	
Gender			0.8
Female	112 (48%)	58 (47%)	
Male	119 (52%)	65 (53%)	
Race			> 0.9
White	197 (85%)	103 (84%)	
Black	30 (13%)	18 (15%)	
Other	4 (1.7%)	2 (1.6%)	
Smoking status			0.4
Never	115 (50%)	68 (55%)	
Former	83 (36%)	35 (28%)	
Current	33 (14%)	20 (16%)	
Frailty			0.9
Low Risk	137 (59%)	73 (59%)	
Intermediate Risk	62 (27%)	35 (28%)	
High Risk	32 (14%)	15 (12%)	
ADI National rank	59 (40, 75)	80 (55, 93)	< 0.001
High ADI	27 (12%)	46 (37%)	< 0.001
BMI category	29.9 (26.8, 34.0)	29.8 (26.0, 33.3)	0.4
Normal weight	35 (15%)	22 (18%)	
Underweight	0 (0%)	1 (0.8%)	
Overweight	82 (35%)	40 (33%)	
Obese	114 (49%)	60 (49%)	
Levels of Fusion			0.6
4–8	217 (94%)	118 (96%)	
8–12	9 (3.9%)	2 (1.6%)	
> 12	5 (2.2%)	3 (2.4%)	
Surgical Approach Type			0.8
Combined	65 (28%)	36 (29%)	
Posterior	166 (72%)	87 (71%)	
Insurance Category			0.9
Private	73 (32%)	41 (33%)	
Public	157 (68%)	81 (66%)	
Indigent/Self Pay	1 (0.4%)	1 (0.8%)	
Any 30-day Readmission*	38 (16%)	22 (18%)	0.7
Readmission 30-day Etiologies**			
Medical	13 (5.6%)	4 (3.3%)	0.3
Weakness/Pain	6 (2.6%)	10 (8.1%)	0.017
Wound/Infection	20 (8.7%)	8 (6.5%)	0.5
Any Reoperation within One Year*	42 (18%)	32 (26%)	0.084
Reoperation reasons within one year**			
CSF/Pseudomeningocele	2 (0.9%)	3 (2.4%)	0.3
Instability/Persistent symptoms	23 (10.0%)	17 (14%)	0.3
Post-op deformity	9 (3.9%)	1 (0.8%)	0.2
Infection/Wound Revision	7 (3.0%)	11 (8.9%)	0.016
Other	2 (0.9%)	3 (2.4%)	0.3
Time to Reoperation	62 (28, 186)	36 (15, 160)	0.2

^*1*^ n (%); Median (Q1, Q3)

^*2*^ Wilcoxon rank sum test; Pearson’s Chi-squared test; Fisher’s exact test; Wilcoxon rank sum exact test

*Only the first event within time period counted

** subcategories/etiologies counted if they occurred at any point within the time period, with multiples allowed

### BMI distribution by food access status

**Fig. 1:**
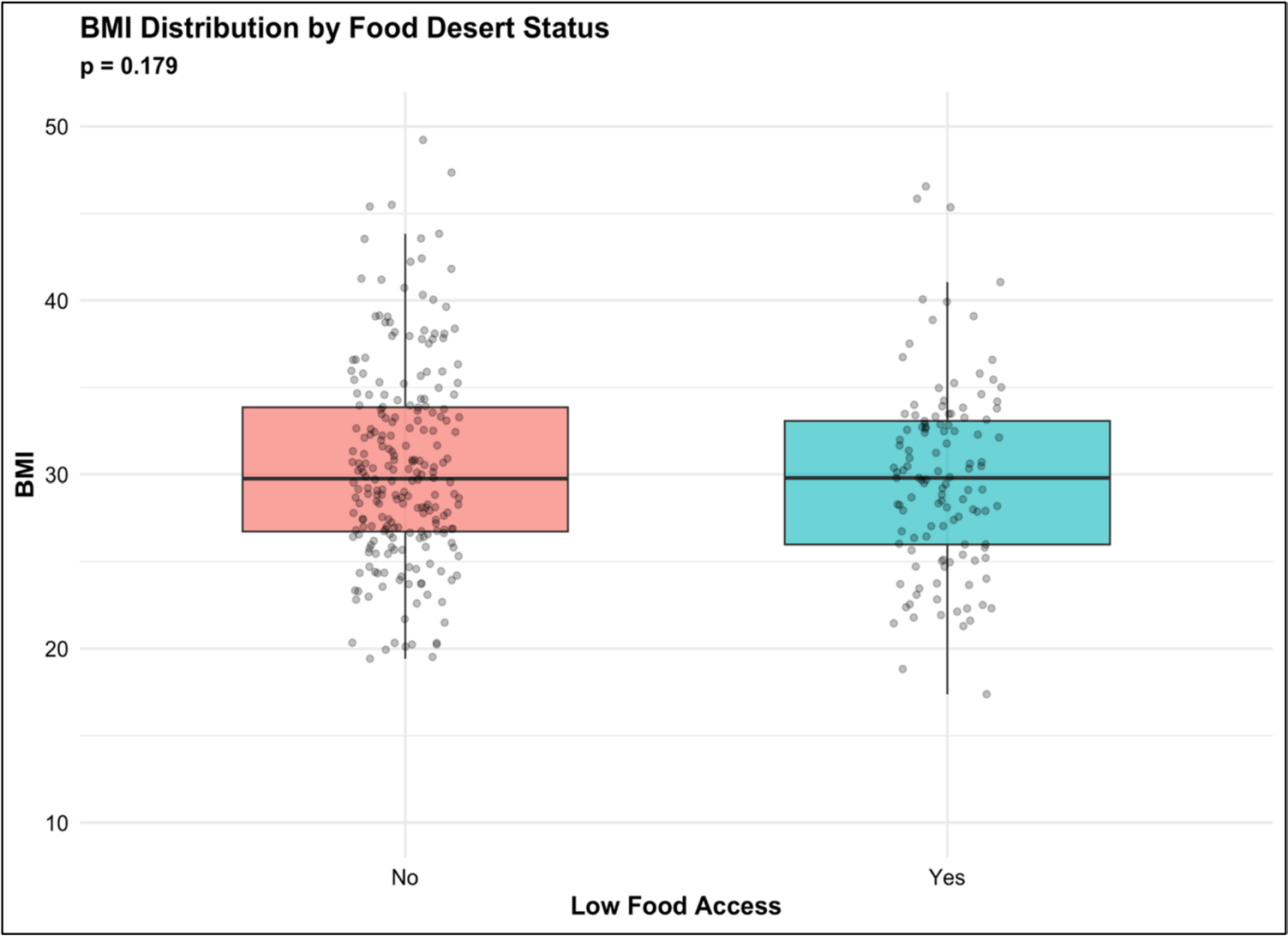
BMI distribution by food desert status

The distribution of body mass index (BMI) categories was comparable between patients residing in low food access regions and those in adequately resourced areas (Fig. 1). In both groups, the majority of patients were classified as overweight (BMI 25–30) or obese (BMI > 30), with relatively few patients categorized as underweight (BMI < 18.5). Statistical analysis revealed no significant difference in BMI distribution between groups (*p* = 0.18), suggesting similar baseline BMI profiles irrespective of food access status.

### Primary outcomes

**Fig. 2:**
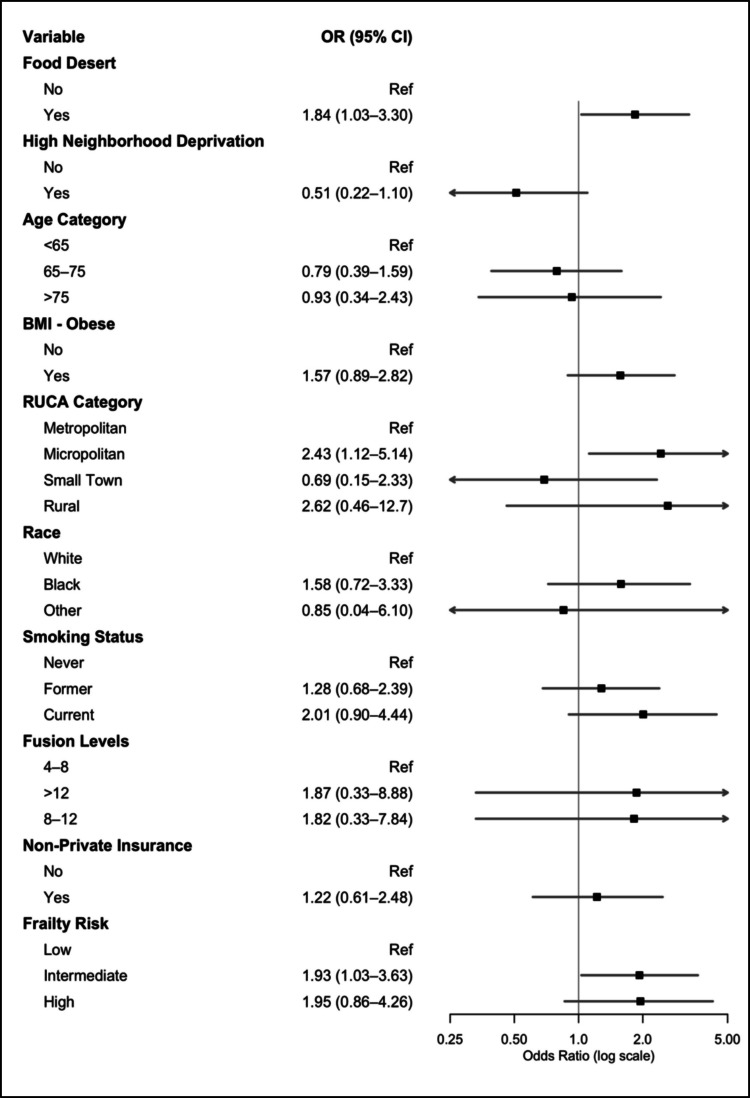
Forest plot for multivariate logistic regression for reoperation

On multivariate logistic regression controlling for age, neighborhood disadvantage, BMI, rurality, race, smoking status, levels of fusion, insurance type, and frailty, patients who resided in a food desert had increased odds of reoperation within one year (OR 1.84, 95%CI 1.03–3.30) (Fig. 2).

**Fig. 3:**
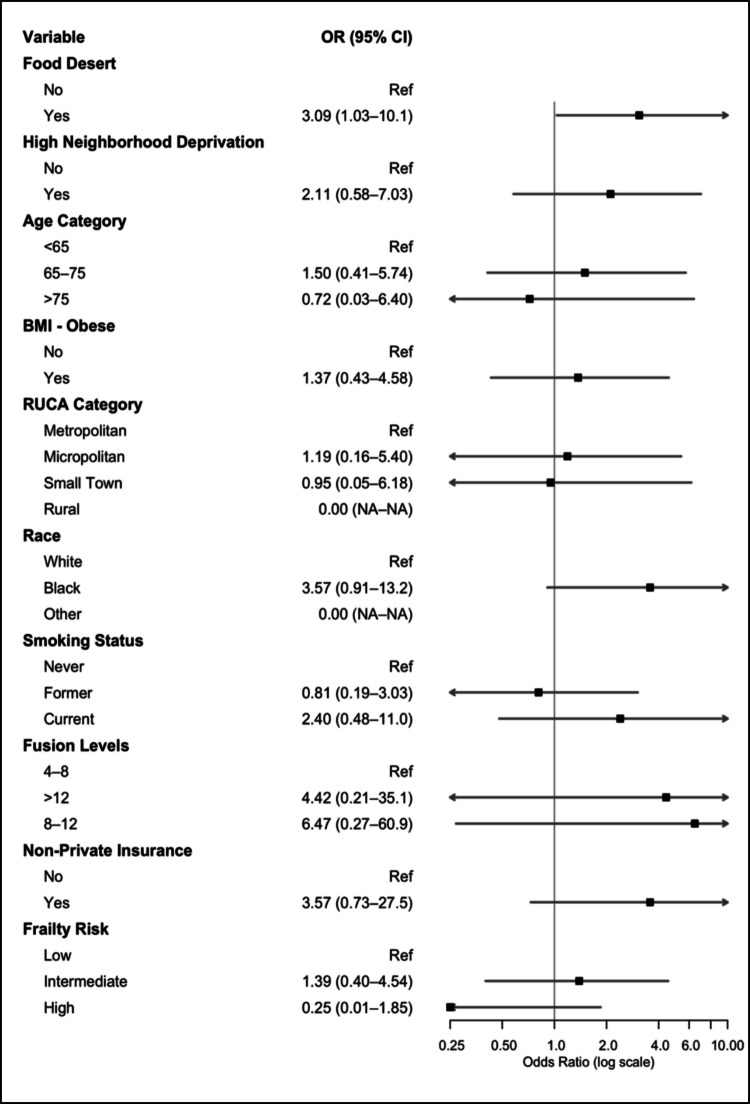
Forest plot for multivariate logistic regression for 30-day readmission due to infection/wound issues

This model had a AUROC of 0.71, indicating moderate discrimination ability. In a separate multivariate logistic regression, patients who lived in a food desert had independently increased odds of readmission within 30 days due to infectious or wound complications (OR 3.09, 95%CI 1.03–10.1) (Fig. 3).

This model had an AUROC of 0.81, indicating good discrimination ability (Supplemental Digital Content, Fig. 2S). These findings were robust upon sensitivity analysis, and no significant collinearity was observed (Supplementary Digital Content, Table S2- S3, Figure S3- S4).

### Time to reoperation

**Fig. 4:**
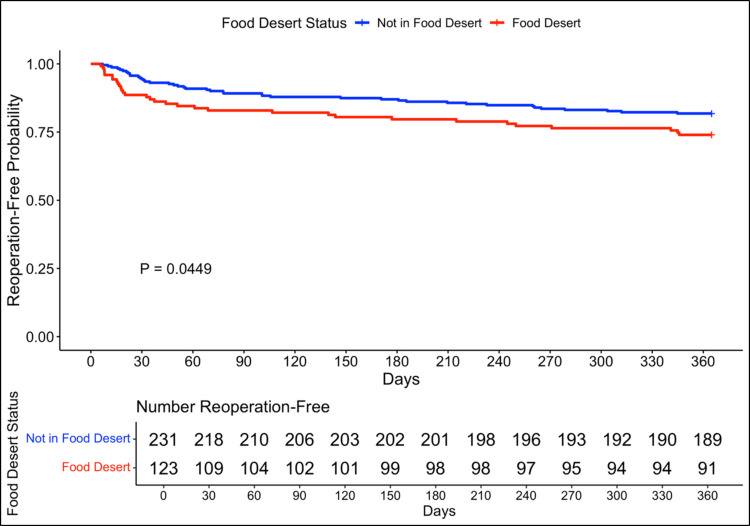
Kaplan Meier survival analysis for reoperation within one year

The median time to reoperation was 51 days (IQR 19–181). In a Fleming Harrington weighted survival analysis model, patients who lived in a food desert had sooner time to reoperation (median time to reoperation: 36 days, IQR (15–160) within one year compared to patients who did not (median time to reoperation 62 days (IQR 28–186) (*p* =. 045) (Fig. 4).

## Discussion

Our results demonstrate that residence in a food desert may be a risk factor for increased risk of infection/wound breakdown and reoperation within one year. Residence in a food desert has been shown to portend worse health outcomes in larger general population studies, but its impact in spine surgical populations has not been characterized.

Complications and reoperation rates remain high in long-segment thoracolumbar fusions, necessitating a deeper understanding of the clinical and social factors that affect outcomes following multi-segment lumbar fusion [42]. Previous literature has focused on nutrition, smoking cessation, and bone density as clinical benchmarks of preoperative optimization for complex multi-level fusions. While our understanding of clinical comorbidities and preoperative optimization has improved, the influence of social factors on outcomes following long segment fusions remains under-explored.

Recent studies have highlighted socioeconomic and racial disparities in access to and outcomes following adult spinal deformity correction, influencing reoperation rates and other complications following lumbar spinal fusion [16, 25, 29, 34]. However, these observations offer more limited insights into the mechanisms of disparity and thus remain limited in identifying intervention strategies.

The importance of food and nutrition in health outcomes is established. An analysis by Azap et al. [6] found that food insecurity was associated with greater odds of readmission and complications in patients who underwent bowel resection for colorectal cancer. Beyond surgical outcomes, Ahern et al. [4] examined the relationship between food access and health outcomes in the general population, finding that low-quality food access was independently linked to higher rates of mortality and diabetes. Further supporting these associations, a review by Gunderson and Ziliak et al. [18] demonstrated that food insecurity was associated with increased rates of depression, asthma, iron deficiency anemia, hypertension, and restriction in activities of daily living (ADL). Together, these findings suggest an at-risk phenotype characterized by limited access to food services, which may contribute to worse health outcomes and postoperative complications, emphasizing the need to address socioeconomic disparities in patient care.

### Food desert status and nutrition

These results suggest residence in a food desert may potentially be associated with poor nutritional quality in spine surgical populations as well. In the context of long-segment spinal fusion, reliable access to adequate nutrition is particularly crucial due to the complex recovery process required for wound healing, successful arthrodesis, and overall post-operative rehabilitation [6, 21, 29, 34].

However, it is important to note that residence in a food desert may not be associated with poor outcomes via its association with decreased BMI or malnutrition; existing literature highlights the association of poor nutritional quality rather than malnutrition with residence in a food desert [13]. Though our analysis found the distribution of BMI to be similar across patients living in and without food deserts, several large-scale studies have found that individuals residing in low food access environments are actually more likely to experience obesity, make poor dietary choices, and consume processed and fast foods that are high in calories but deficient in essential proteins, vitamins, and minerals necessary for bone regeneration and wound healing [4, 13, 18]. Evidence suggests that in both urban and rural settings, overall quality of nutrition rather than density of calories drives many of the observed health disparities in today’s communities [17, 39]. It is possible that poor nutritional quality rather than malnutrition or BMI associated with residence in a food desert may be an underlying driver of poor outcomes in long segment fusion.

Dietary quality deficiencies may contribute to impaired fusion and structural integrity in addition to superficial wound healing processes. This is supported by a body of literature highlighting the importance of micronutrients in optimizing surgical recovery [36]. Poor nutritional status has additionally been independently linked to higher odds of adverse events, readmission, and delayed wound healing following lumbar spine fusion and resection of spinal metastases [7, 11]. These findings suggest that integrated nutritional and social support strategies in patients undergoing long-segment lumbar fusions may warrant future investigation.

It is also possible that residence in a food desert may serve as an additional surrogate for area-level socioeconomic status. As both indices are constructed from ACS data, there may be some degree of overlap. However, food desert status specifically focuses on access to grocery stores, estimated distances between stores and residences using geospatial and road analysis, as well as the relative ability of residents to reach these spaces through vehicular access and poverty rate, integrating not only infrastructure disparities but also individual barriers, while area-level deprivation generally is built upon employment, income, racial disparities, and housing quality. Despite this, it is possible that access to food may capture similar variables, which may account for some degree of the observations seen in this analysis.

### Future directions and utility of food deserts

Identifying socioeconomic risk factors and modifiable drivers of health disparities in long-segment fusion surgery remains challenging. However, evidence-based strategies aimed at addressing the SDoH have long been established in other surgical subspecialties, including transplant and colorectal surgery.

As morbidity rates in long segment thoracolumbar fusion remain significant, there is a need to adopt evidence-based strategies that address SDoH and support patient optimization, similar to approaches successfully implemented in other high-risk surgical subspecialties [27, 30]. Many of these specialties have implemented screening tools targeted at identifying patients with food insecurity [36]. Our results suggest that food desert status may offer an easily quantifiable capture of a patient’s built environment that may assist in identifying high-risk socio-environmental phenotypes. These rapidly identifiable patients may be targeted with interventions to optimize outcomes.

Nutritional prehabilitation in surgical patients is well established and has gained significant traction in other specialties, becoming a standardized process in many surgical fields [15, 20]. In the PREHAB trial published by Molenaar et al. [30], patients undergoing colorectal cancer surgery were allocated to either a comprehensive prehabilitation program-encompassing exercise, nutrition, psychological support, and smoking cessation-or to standard care. Patients undergoing prehabilitation were less likely to have postoperative medical and surgical complications 30 days after surgery. Existing studies have also identified that focused nutritional supplementation programs in the perioperative period in spine surgery have also been found to improve outcomes [9, 33]. These interventions focused on this high-risk population may ultimately improve disparities in postoperative outcomes. System-wide recognition of the role of social factors in impacting post-operative course and establishment of more community-level resources may also be beneficial in improving outcomes [22].

Utilizing measures of a patient’s built environment, such as residence in a food desert, integration of evidence-based preoperative nutritional optimization, and social support resources for at risk patients into care pathways may help reduce disparities and enhance postoperative outcomes. Future research should explore targeted interventions and policy-driven solutions to enhance nutritional access and equity in spine surgery.

## Limitations

This study is limited in its design as a single-center retrospective analysis and its associated biases. Given the association of the investigated variable on state-level demographics, infrastructure, and funding, these results may not be generalizable to other states. While we controlled for key demographic and clinical factors, residual confounding may still exist. Residence in food deserts may underestimate true nutritional risk, as food insecurity can exist within areas classified as having adequate food access. Additionally, we were unable to assess direct nutritional markers such as serum albumin or preoperative dietary intake, which could provide a more granular understanding of the relationship between nutrition and surgical outcomes. It is possible that food desert residence may be a marker for poor overall health as well, though adjustment for clinical frailty was conducted in our multivariate models. Fusion status, determined by radiographic evidence, was not consistently available and thus could not be analyzed. While our findings suggest a strong association between low food access and increased reoperation rates, causality cannot be established, and future prospective studies are planned in an effort to validate these results and explore potential intervention strategies. It is important to recognize that food desert status may be a surrogate for overall socioeconomic deprivation. Though we adjust for potential confounding by controlling for established, validated markers of socioeconomic status in our analysis, the potential for interaction should be recognized. However, our results are the first demonstration of the association of residence in a food desert with poor postoperative outcomes and highlights the utility of area-level measures of patient SDoH in risk prognostication.

## Conclusion

Residence in a food desert may be associated with increased reoperation rates following long-segment lumbar fusion, underscoring the critical role of nutrition in surgical recovery. Patients with who live in food deserts likely face challenges in achieving optimal nutritional status, which may contribute to delayed wound healing, poor bone health, and increased complications requiring reoperation.

## Supplementary Information

Below is the link to the electronic supplementary material.

ESM 1 Supplementary Material 1 (DOCX 187 KB)

## Data Availability

Data can be made available upon reasonable request.

## Abbreviations

ADI: Area Deprivation Index
BMI: Body Mass Index
CMS: Centers for Medicare/Medicaid Services
EMR: Electronic Medical Record
FIPS: Federal Information Processing Standard
HFRS: Hospital Frailty Risk Score
HRSA: Health Resources Services Administration
SDoH: Social Determinants of Health
SVI: Social Determinants of Health
USDA: United States Department of Agriculture
