# Supplementary material for: The association of food deserts with readmission and re-operation following long-segment lumbar fusion
Source: Acta Neurochir (Wien). 2026 Apr 11;168(1):106. doi: 10.1007/s00701-026-06823-5 (PMC13183740; doi:10.1007/s00701-026-06823-5)
Supplement: Supplementary file 1 — Supplementary Material 1 (DOCX 187 KB) [file 701_2026_6823_MOESM1_ESM.docx]

**Supplementary Digital Content**

**Table S1.** Distribution of Reoperations

| **Characteristic** | **Overall** |
| --- | --- |
|  | N = 354*^1^* |
| Reoperate within one year | 74 |
| Reoperate from 0-90 days | 46 (62.2%) |
| Reoperate from 91-365 | 33 (44.6%) |
| *^1^* n (%) |  |

**Table S2.** Variance inflation factors for reoperation model

|  | GVIF | Df | GVIF^(1/(2*Df)) |
| --- | --- | --- | --- |
| Food Desert | 1.135584 | 1 | 1.065638 |
| Gender | 1.105388 | 1 | 1.051374 |
| Neighborhood Deprivation | 1.283474 | 1 | 1.132905 |
| Age | 1.741337 | 2 | 1.148737 |
| BMI | 1.151572 | 1 | 1.073113 |
| Rurality | 1.310257 | 3 | 1.046067 |
| Race | 1.190689 | 2 | 1.044599 |
| Smoking Status | 1.387829 | 2 | 1.085385 |
| Fusion levels Category | 1.11979 | 2 | 1.028689 |
| Insurance | 1.473178 | 1 | 1.213746 |
| Frailty | 1.197851 | 2 | 1.046166 |

**Table S3.** Variance inflation factors for readmission model

|  | GVIF | Df | GVIF^(1/(2*Df)) |
| --- | --- | --- | --- |
| Food Desert | 1.090639 | 1 | 1.044337 |
| Gender | 1.138618 | 1 | 1.06706 |
| Neighborhood Deprivation | 1.162568 | 1 | 1.078224 |
| Age | 1.580843 | 2 | 1.121301 |
| BMI | 1.161847 | 1 | 1.07789 |
| Rurality | 1.195549 | 3 | 1.030215 |
| Race | 1.261624 | 2 | 1.059821 |
| Smoking Status | 1.552143 | 2 | 1.116177 |
| Fusion levels Category | 1.294271 | 2 | 1.066612 |
| Insurance | 1.308645 | 1 | 1.14396 |
| Frailty | 1.327968 | 2 | 1.073487 |


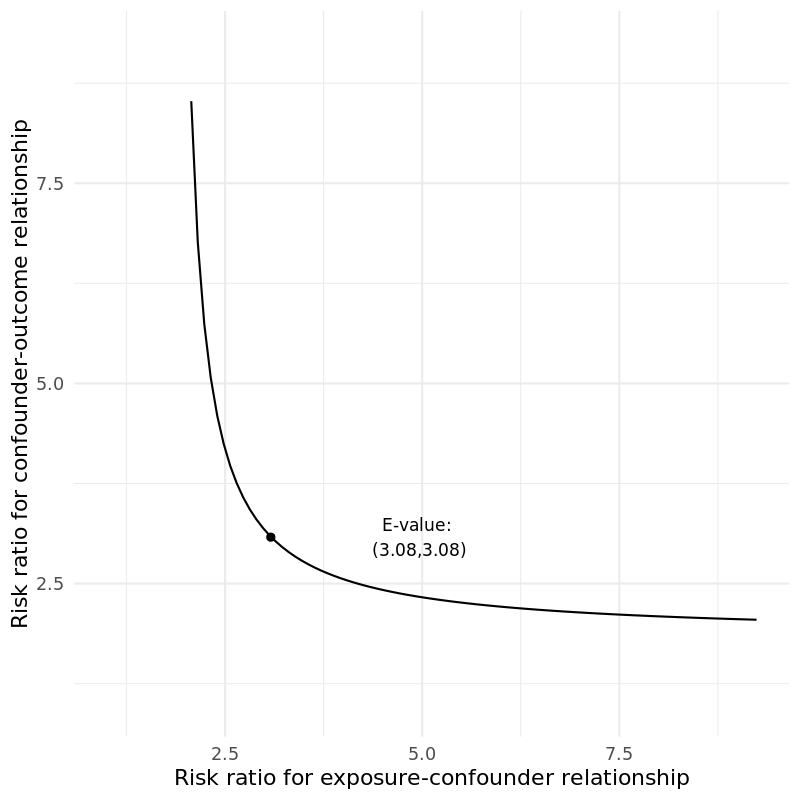


**Figure S1.** E-value for reoperation


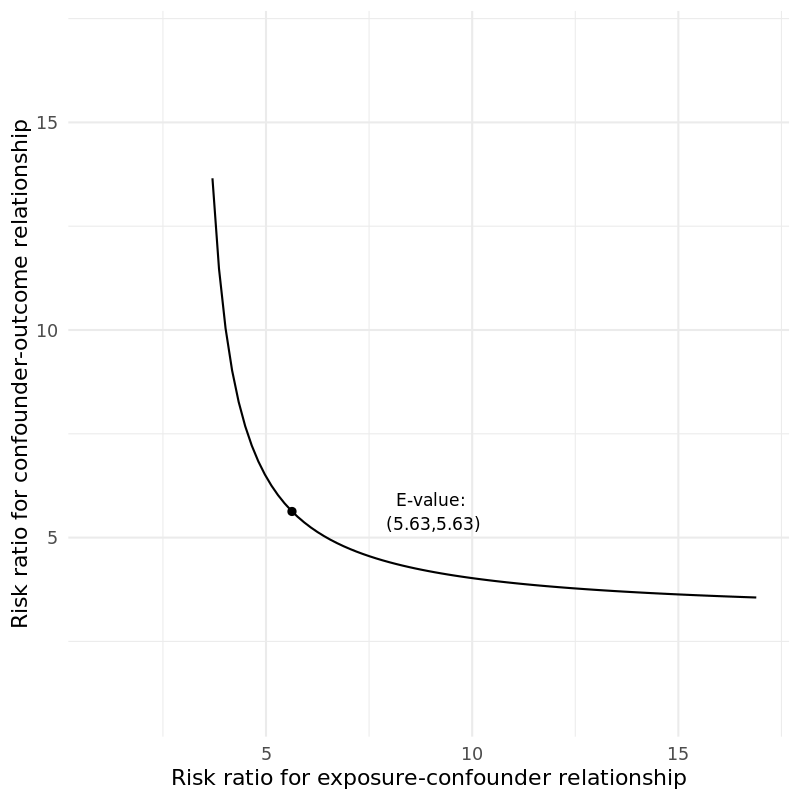


**Figure S2.** E-value for readmission due to infection/wound


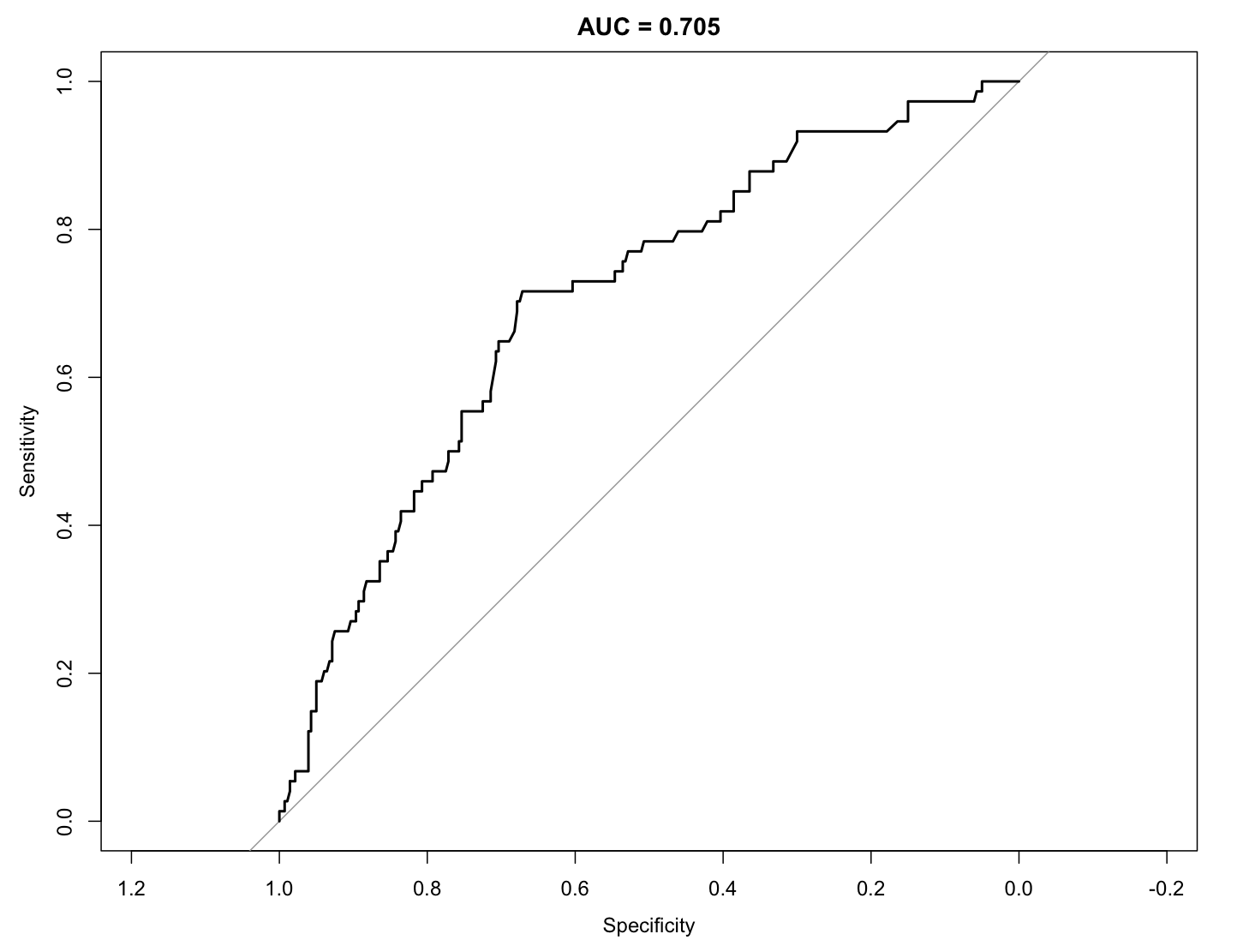


**Figure S3.** AUROC for reoperation model


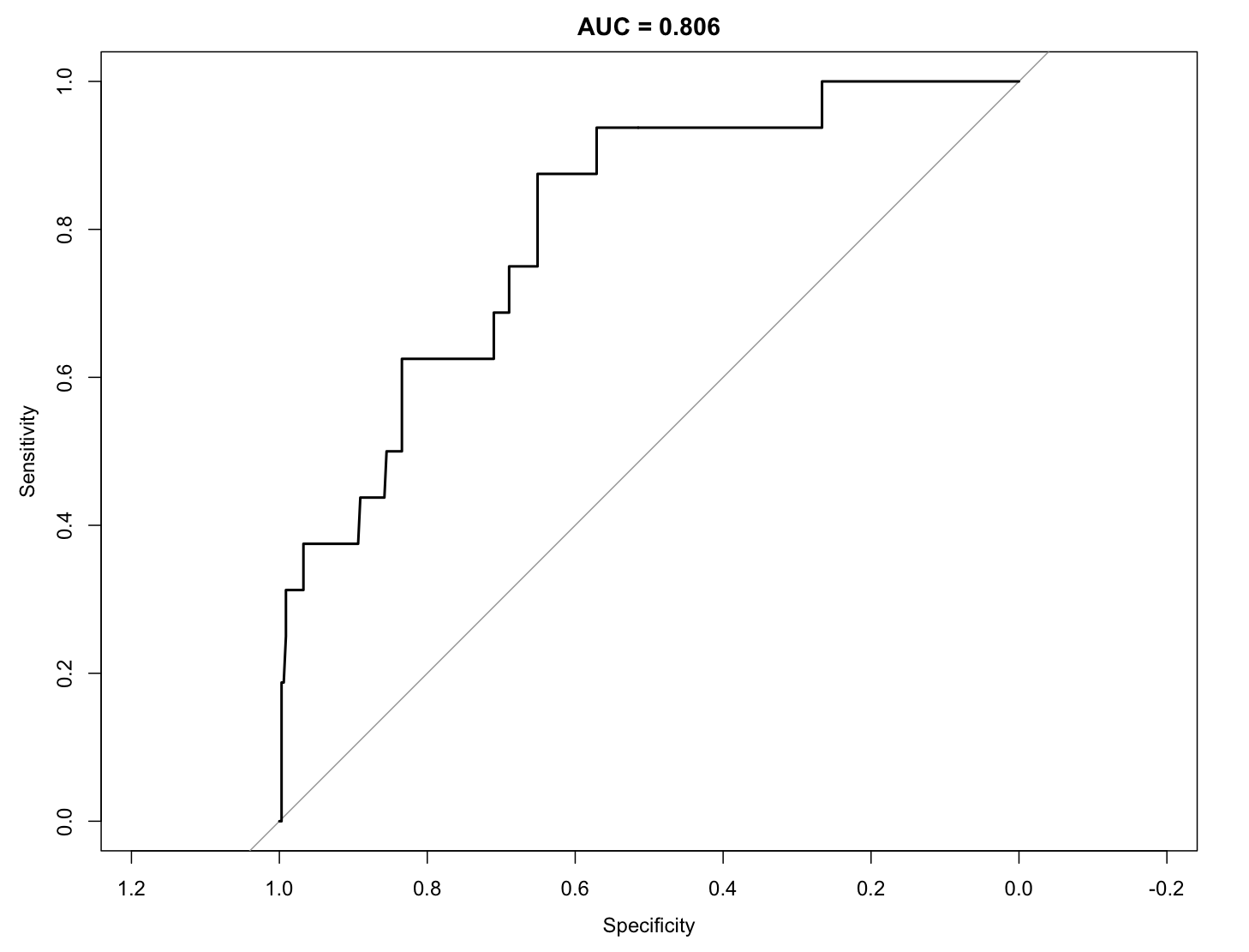


**Figure S4.** AUROC for readmission due to infection/wound
